# From Emails to EMR: Implementing I-PASS Among Inpatient Palliative Care Clinicians at a Comprehensive Cancer Center—A Quality Improvement Initiative

**DOI:** 10.3390/cancers17172875

**Published:** 2025-09-01

**Authors:** Jaya Amaram-Davila, Maria Franco Vega, Patricia Bramati, Holly Stewart, Monica Aceves, Shalini Dalal, Akhila Reddy, Ahsan Azhar, Suresh K. Reddy, Diane C. Bodurka, Marina George, Mohamed Ait Aiss, Eduardo Bruera

**Affiliations:** 1 Department of Palliative, Rehabilitation, and Integrative Medicine The University of Texas MD Anderson Cancer, Houston, TX 77030, USA; 2 Department of Hospital Medicine, The University of Texas MD Anderson Cancer, Houston, TX 77030, USA; 3 Division of Education & Training, The University of Texas MD Anderson Cancer, Houston, TX 77030, USA

**Keywords:** handoff, structured, safety, efficiency, provider satisfaction, palliative care

## Abstract

Background: Inpatient palliative care consultation services operate with an interdisciplinary team, where effective handoffs are crucial for coordinated patient care. We aimed to replace encrypted email handoffs with a more concise and uniform handoff using I-PASS (illness severity, patient summary, action list, situational awareness, contingency planning, and synthesis by receiver) integrated within the electronic medical record (EMR). Aim and Measures: Within six months of launch, our goal was to achieve 90% I-PASS utilization for hospitalized acutely ill patients with cancer receiving palliative care consultation. Intervention: In January 2021, our quality improvement team, consisting of physicians, advanced practice providers, and trainees, began implementing I-PASS using the plan–do–study–act cycle. After providing training sessions for all palliative care clinicians, I-PASS went live on October 1, 2021. I-PASS utilization was tracked via random and monthly audits of EMRs. Through anonymous surveys, both pre- and post-implementation, we gathered clinician feedback and concerns about the handoff system. Survey responses were compared using the Mann–Whitney test. Outcomes: Within six months of implementation, the I-PASS utilization rate reached > 99%. The survey participation rates were 70% (45/64) and 82% (49/60) for the pre-and post-implementation periods, respectively. Respondents provided answers on one to five scale (mean, standard deviation, SD): lower accuracy with email (3.53, SD = 0.98) vs. I-PASS (4.20, SD = 0.83), *p* < 0.001; handoff lengthier with email (4.17, SD = 1.05) vs. I-PASS (2.1, SD = 1.15), *p* < 0.001; the time required was longer with email (3.0, SD = 1.22) vs. I-PASS (1.71, SD = 0.73), *p* < 0.001. Overall, respondents found I-PASS to be significantly better (4.69, SD = 0.58). Conclusion: I-PASS was fully adopted by the team, with nearly 100% utilization and strong clinician endorsement as an effective communication tool. Future efforts should focus on optimizing usability, particularly by educating clinicians on smartphone EMR access and enabling the timely and streamlined editing of I-PASS.

## Simple Summary

What is already known on this topic: A clear and concise handoff between team members is essential for safe and effective patient care. I-PASS, a validated handoff system that is integrated within the electronic medical record (EMR), has been found to reduce medical errors resulting from communication gaps. There is, however, insufficient information on whether I-PASS adaptation can improve communication among consultation services, such as palliative care providers who work in an interdisciplinary fashion. What this study adds: The focus of this quality improvement initiative was to transition from the encrypted email handoff process to a more uniform and concise I-PASS format within EMRs shared among all interdisciplinary team members. Our transition improved provider satisfaction, streamlined access to patient information, and facilitated more efficient and timely coordination of clinical care between the clinical team members. How this study might affect research, practice, or policyv variability: in the handoff process persists. Our approach to transitioning from an encrypted email handoff to I-PASS can be applied to various subspecialties that function in an interdisciplinary fashion. Through a timely, uniform, and accurate patient care handoff, clinicians can work more cohesively, enhancing patient care and reducing provider burnout.

## 1. Introduction

Patients with advanced cancer often face a wide range of physical, psychosocial, and spiritual symptoms [1,2,3,4]. To manage such a complex symptom burden, our inpatient palliative care consult service operates in an interdisciplinary team fashion, focusing its discussion to address a wide variety of symptoms, including advance care planning [3,4,5]. Miscommunication among the team members during clinical rounds or a shift change can lead to serious medical errors. A prospective intervention study by Starmer et al. identified 5516 medical errors among 10,740 patient admissions, all related to improper handoff communication [6]. They observed a marked reduction in the overall medical error rate (23%) and preventable adverse event rate (30%) due to the introduction of a standardized handoff process [6]. A real-time exchange of information among team members is crucial and should be facilitated by a structured and standardized handoff process enabling timely and effective patient care coordination [6,7,8,9,10].

Previously, our palliative care team used an encrypted group email to conduct patient handoffs. However, as demand for palliative care services grew [1,5]—along with patient volume and team size—the email-based handoff process became increasingly inconsistent, leading to redundant information and delays in care communication, making it tedious to locate necessary patient care updates. In 2016, our institution adopted a new electronic medical record (EMR) system, called EPIC, that has an embedded handoff tool, I-PASS (Illness severity, Patient summary, Action list, Situational awareness, contingency plan, and Synthesis by the receiver). I-PASS is widely used across healthcare systems in the United States and has been reported to increase patient safety, enhance provider communication, and reduce handoff-related errors [6,7,8,9,10].

To enhance timely and coordinated patient care, our palliative care team launched a quality improvement (QI) initiative transforming inpatient handoffs from emails to an EMR-based I-PASS system—creating a standardized, efficient, and user-friendly communication platform that saves time and strengthens care delivery.

## 2. Materials and Methods

### 2.1. Quality Improvement Team

In January 2021, we formed a QI team consisting of 10 team members, which included 3 palliative care physicians, 2 palliative care advanced practice providers, 2 hospice and palliative care trainees, 1 institutional I-PASS physician champion, and 1 operational manager. The QI team developed a plan–do–study–act cycle to initiate the transition from email handoffs to I-PASS. The institutional Quality Improvement and Assessment Board approved this project.

### 2.2. Study Participants

All supportive and palliative care service clinicians participated in this QI project. At the time of this project initiation, our palliative care team consisted of 25 physicians trained in hospice and palliative care, 22 advanced practice providers, 10 hospice and palliative care trainees, 3 psychologists, 4 counselors, and 2 clinical pharmacists. Other ancillary services such as chaplaincy, social work, and case management were available on an as-needed basis [1].

### 2.3. Inpatient Palliative Care Consultation Structure

The inpatient palliative care consultation service comprised seven teams. Each team typically consisted of one attending physician, two to three advanced practice providers, and one palliative care trainee. Clinical pharmacists and psychologists provided support across all teams. A typical weekday involved approximately 27 to 30 palliative care clinicians offering consultation services to 150–180 acutely ill hospitalized patients with cancer.

### 2.4. E-Mail Handoff Process

**Figure 1:**
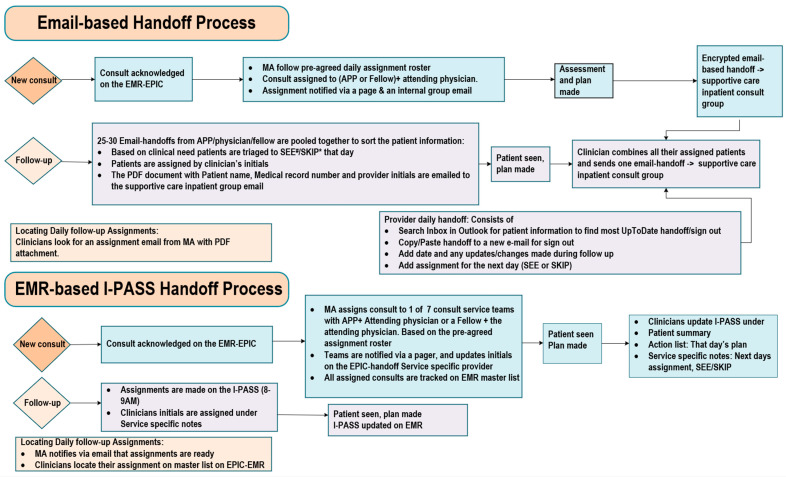
Workflow map of the e-mail handoff and I-PASS handoff process. Workflow comparison of email- vs. EMR-based I-PASS handoffs. In the email-based system, MAs assign consults, and clinicians send handoff emails (plan, code status, social context, SEE/SKIP). Follow-ups are assigned by the charge clinicians based on previous emails. The PDF of the assignments is then distributed to the teams. In the EMR-based I-PASS, MAs assign consults, clinicians document in I-PASS (summary, action list, notes, SEE/SKIP), and charge clinicians finalize assignments in the EMR with MA notification to the team. Note: EMR: electronic health record; MAs: medical assistants. # SEE: patients assigned to see that day, especially those with high symptom burden and need for medication adjustments. * Skip: patients with low symptom burden but still admitted for acute illness are seen every 3–4 days. +I-PASS: illness severity, patient summary, action list, situational awareness, contingency plan, and synthesis by the receiver.

**Figure 2:**
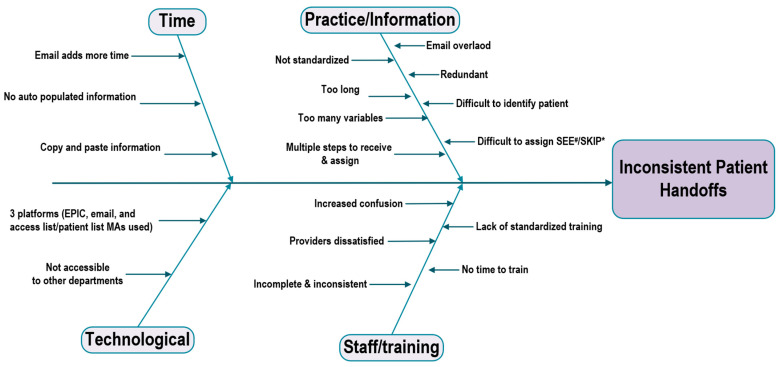
A fishbone diagram illustrating barriers that cause an inconsistent handoff process. Note: EPIC, electronic medical record; MAs: medical assistants. # SEE: patients scheduled to see that day, especially those with high symptom burden and need for medication adjustments. * Skip: patients with low symptom burden but still hospitalized are seen every 3–4 days.

We created a process flow map to streamline the consultation process, from receiving a palliative care consultation request to updating the email handoff (Figure 1). The old handoff process involved each palliative care clinician from each consultation team preparing an email handoff for their assigned patients. The email handoff summarized the clinical status, symptom management plans, psychosocial concerns, and recommended next steps. These encrypted email handoffs were shared daily in a common pool accessible to all palliative care clinicians, ensuring that the team scheduled for the next day was informed of the patient’s status, resulting in approximately 25–28 emails per day. During working hours, there was one palliative care physician and an advanced practice provider, known as charge clinicians, who handled urgent patient care issues. These charge clinicians reviewed all 25–28 email handoffs to create the patient assignment list. The inconsistency among email handoffs made this review process time-consuming and caused delays. Figure 2 illustrates the root cause analysis of obstacles and challenges associated with email handoffs, including variability in the handoff process, lengthy and multiple emails, and frequent copying and pasting from progress notes.

### 2.5. I-PASS Handoff Interventions

The QI team introduced a step-by-step transition from an email-based handoff process to a clear, concise, and standardized EPIC-integrated I-PASS handoff system. The project aimed to reach at least 90% utilization of daily I-PASS handoffs among acutely ill patients with cancer seen by the palliative care consult service within six months of rollout.

The QI team adapted the I-PASS framework to cover key aspects of patient care, including symptom assessment, pain status, opioid regimens, opioid risk assessments, psychosocial factors, goals of care, and code status. In collaboration with the EPIC information technology team, smart phrases were created to auto-fill essential fields, ensuring consistency and efficiency. All palliative care clinicians received one-on-one coaching and access to step-by-step guides, followed by a mock trial to ensure they were comfortable with the system. I-PASS was launched on 1 October 2021.

### 2.6. Data Collection and Compliance Monitoring

The QI team conducted scheduled monthly and random audits through the inbuilt data system on the EPIC platform to monitor I-PASS utilization. We obtained real-time feedback from clinicians to improve adaptability. Baseline compliance could not be assessed because pre-implementation handoffs were conducted through unstructured encrypted emails. I-PASS compliance was tracked prospectively from its implementation through the first six months of its launch.

### 2.7. Palliative Care Provider Handoff Survey

An anonymous pre- and post-implementation survey assessed palliative care clinicians’ perceptions of the email handoff process and the I-PASS, respectively. Questions covered the ease of locating patient information, handoff accuracy, the length of handoffs, the presence of redundant information, the time needed to update handoffs, the ease of triage, and overall satisfaction. Most responses used a 5-point Likert scale (1 = strongly disagree to 5 = strongly agree). One question measured the time to complete handoffs per patient (1 = less than 2 min, 2 = 2–4 min, 3 = 5–10 min, 4 = 11–20 min, 5 = more than 20 min), and another compared satisfaction with I-PASS versus email handoffs (1 = much worse to 5 = much better). To ensure validity, the survey was pretested with five team members (two physicians and three advanced practice providers), and revisions were made accordingly. The final pre- and post-implementation surveys included 13 and 15 items, respectively. The estimated time to complete the survey was approximately 3 min. To create a safe space and increase participation, the surveys were anonymized. The pre-implementation survey was sent to 64 and the post-implementation survey was sent to 60 palliative care clinicians employed in the palliative care department at the time. Four counselors opted out of the post-implementation survey. The survey was administered via Qualtrics, with automatic weekly reminders provided over a four-week period for those who did not complete the survey. A small number of clinicians who expressed hesitancy about using I-PASS were given time to adapt to the new system. Our QI team met with them in person to understand their concerns and used their feedback to improve the I-PASS workflow.

### 2.8. Statistical Analysis

Monthly I-PASS utilization rates were visualized using a scatterplot. A box plot was created to compare utilization rates during the first and second halves of the intervention. The pre- and post-implementation survey responses were analyzed by calculating the mean and standard deviation for each question. The Mann–Whitney test was used to compare the two groups; a *p*-value < 0.05 was considered significant. The data were analyzed using Minitab 18 statistical software (Minitab, LLC Herndon, VA, USA).

## 3. Results

**Figure 3:**
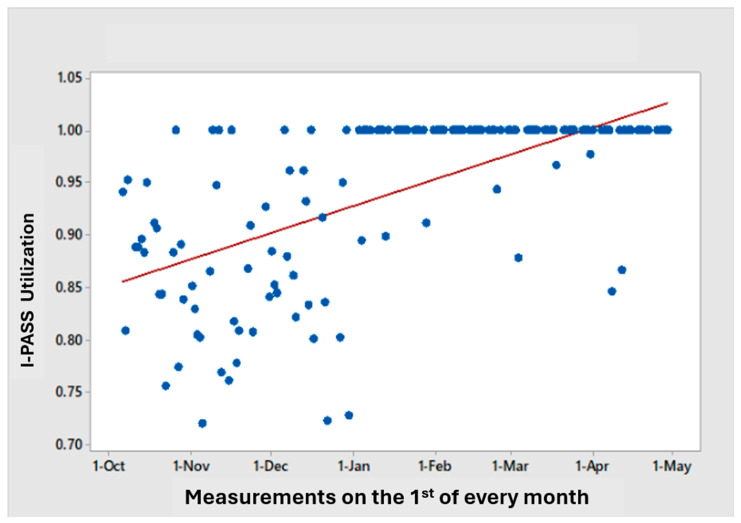
Each blue dot represents a monthly handoff observation measured for I-PASS compliance. Compliance displayed wide variability in the early months (October-December, 2021), which progressively narrowed over time (January-March, 2022)—rising from variable mid-80% utilization at implementation in October 2021 to over 98% by March 2022—demonstrating improved consistency and sustained, system-wide adherence.

**Figure 4:**
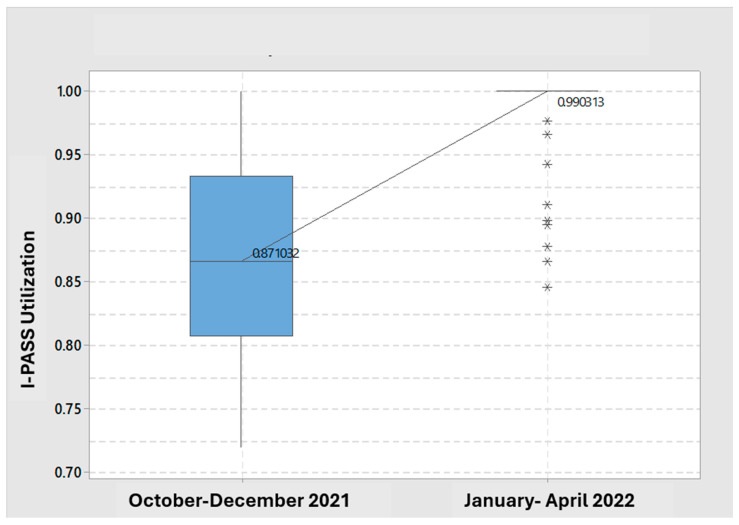
Shows the distribution of I-PASS utilization, increasing from 87% in the first half of implementation (October–December 2021) to 99% in the second half (January–April 2022). By March 2022, utilization exceeded the target of >90% for daily handoffs among palliative care consult patients. * *p* < 0.05.

We monitored I-PASS utilization on weekdays, including approximately 100–110 patient encounters/day and 3000 patient encounters/month, excluding weekends and holidays, due to limited clinician availability. As shown in Figure 3, each blue dot represents an individual handoff observation measured for I-PASS compliance. Compliance widely varied in the early months, which progressively narrowed over time—rising from variable mid-80% utilization at implementation in October 2021 to over 98% by March 2022—demonstrating improved consistency and sustained, system-wide adherence. Figure 4 shows the distribution of I-PASS utilization, increasing from 87% in the first half of implementation (October–December 2021) to 99% in the second half (January–April 2022). By March 2022, utilization exceeded the target of > 90% for daily handoffs.

### Survey Results

Table 1 summarizes the survey results comparing the pre-implementation (email) phase with the post-implementation (I-PASS) phase. The pre-implementation survey response rate was 70% (45/64). The respondents consisted of 18 physicians, 20 advanced practice providers, 3 trainees, and 4 psychologists. The post-implementation response rate was 82% (49/60), with respondents comprising 21 physicians, 22 advanced practice providers, 6 trainees, and 0 psychologists. In the pre-implementation survey (email handoff), most of the respondents indicated that a change in handoff was needed. Comparing the mean and standard deviation (SD) scores on survey responses, the respondents expressed handoff accuracy was lower with email (M = 3.53, SD = 0.98) vs. I-PASS (M = 4.20, SD = 0.83), *p* < 0.001; handoff was lengthier with email (M = 4.17, SD = 1.05) vs. I-PASS (M = 2.1, SD = 1.15), *p* < 0.001; there was more redundant information in the email handoff (M = 3.78, SD = 1.16) vs. I-PASS (M = 2.45, SD = 1.11), *p* < 0.0001; the time required in minutes to complete handoff per patient was longer with email (M = 3.0, SD = 1.22) vs. I-PASS (M = 1.71, SD = 0.73), *p* < 0.001. Overall, respondents found I-PASS to be significantly better (M = 4.69, SD = 0.58).

## 4. Discussion

In this QI project, we achieved a goal of over 99% utilization with I-PASS among all palliative care clinicians within six months of implementation, demonstrating the feasibility of the initiative. The high and sustained adherence marked a successful transition from the email-based handoff process to EMR-based I-PASS. This project underscores the importance of a concise and uniform handoff process—such as I-PASS—when integrated into EMRs like EPIC. Such practices are especially critical for services such as palliative care, which operate in an interdisciplinary manner to manage complex symptom burden and family dynamics [1,4,5].

The I-PASS utilization rate remained consistently above 90% throughout the study period. Clinicians found daily completion of I-PASS for all their patients to be easy and time-saving. The palliative care service primarily manages severe cancer-related symptoms, with cancer pain being the most common consultation reason [2,11]. Cancer pain management typically includes initiating opioids, monitoring response, adjusting doses for organ dysfunction or uncontrolled pain while monitoring for signs of overdoses and acute pain crisis [12]. They also prescribe opioids at discharge, and coordinate follow-up with the ambulatory palliative care clinic [1]. A high-quality handoff should provide clear details on the patient’s condition, care plan, and next steps to ensure smooth care transitions.

Some patients with cancer carry risk factors for nonmedical opioid use (NMOU), which is defined as using prescription opioids for purposes other than intended. Such high-risk behaviors can lead to safety concerns [13,14]. Effective palliative care handoff for hospitalized patients receiving opioids should include their current opioid regimen, any high-risk factors for NMOU, such as information on positive screening in the CAGE [15] and/or SOAPP [16] questionnaires, a history of alcohol or other substance use disorder, high demands for intravenous opioids, or aggressive behaviors toward staff when demands are unmet [13,14]. Such information can help the cross-cover team prepare, improving clinician and staff safety [17]. Further research is needed to determine additional elements that enhance handoffs for this high-risk population.

Previous studies have supported the notion that the I-PASS handoff system effectively reduces medical errors and delays in care [6,9]. A vital aspect of the I-PASS format is that it has a dedicated area for code status and contingency planning. A proper handoff should include information on the patient’s code status, the name of the medical power of attorney, relevant family dynamics, and any social challenges [5,18]. This is crucial among patients with advanced illnesses such as cancer, as their trajectories change rapidly [18,19,20]. For example, if the palliative care team holds a goals-of-care meeting resulting in a change of code status from "Full code" to "Do Not Resuscitate," documenting this in the handoff enables the night-time cross-covering team to honor the patient’s preferences [18,19]. Such communication reduces response times, facilitates appropriate interventions, and prevents delays in care [20]. The I-PASS handoff effectively conveyed this vital patient information, and our palliative care clinicians highly appreciated timely access to these data through EPIC.

A key strength of our study is identifying barriers to effective communication within a busy inpatient palliative care consultation service. With multiple providers working in an interdisciplinary manner, clinical updates often vary, making it challenging to present a consistent message and potentially compromising patient safety. A consistent, uniform, and concise handoff through the EPIC EMR, utilizing I-PASS, helped address these challenges. High utilization and sustained adherence suggest that I-PASS is both practical and sustainable in the palliative care setting.

This study has two main limitations. First, precise data on daily handoff emails before I-PASS implementation were unavailable; based on clinician assignments and patient distribution, we estimated 25–28 emails per day. Second, baseline compliance could not be measured because pre-I-PASS handoffs were conducted via unstructured emails. Despite these limitations, our findings demonstrate substantial and sustained improvements in compliance and consistency. Further research is needed to assess handoff challenges in palliative care populations at other centers.

## 5. Conclusions

This quality improvement project demonstrated that transitioning handoffs from email to an EMR-integrated I-PASS system in a palliative care consultation service is both feasible and effective—enhancing communication, efficiency, and clinician satisfaction. The shift from email-based handoffs to EMR as exceeded utilization goals, with sustained adherence above 99%. Broader adoption and evaluation across diverse clinical settings are strongly warranted.

## Figures and Tables

**Table 1:** Palliative Care Clinicians’ Perceptions Towards Patient Handoff Communication.

Survey Question on the Handoff Process	Palliative Care Clinicians		
	E-Mail (Pre-Implementation) N = 64; *n* = 45 M: Means (SD^@^)	I-PASS^+^ (Post-Implementation) N = 60; *n* = 49 M (SD)	*p*-Value
I can easily find the patient’s information from the current handoff while rounding on the inpatient service ^a^	3.33 (1.41)	4.45 (0.97)	<0.001
I feel that the current handoff is too long ^a^	4.18 (1.05)	2.10 (1.15)	<0.001
I find that the information is accurate in the current handoff ^a^	3.53 (0.98)	4.20 (0.83)	<0.001
I feel like information is redundant in the current handoff ^a^	3.78 (1.16)	2.45 (1.11)	<0.001
I can easily assess if the patient is a See^#^/Skip* (triaging) from the handoff ^a^	3.00 (1.24)	4.43 (0.93)	<0.001
I take an average of this amount of time per patient to compose my handoff ^b^	2.96 (1.22)	1.71 (0.73)	<0.001
When away from the workstation, I rely on my phone to access handoff information ^a^	4.13 (1.19)	2.76 (1.57)	<0.001
Our current email-handoff system needs a change ^a^	4.27 (1.07)	N/A	N/A
I am satisfied with the timeliness of the information provided on the I-PASS handoff ^a^	N/A	4.20 (0.90)	N/A
Overall, as compared to email, I find the I-pass handoff system ^c^	N/A	4.69 (0.58)	N/A

Notes: @ M: mean; SD: Standard Deviation. ^a^ Likert scale 1–5: 1 = strongly disagree, 2 = somewhat disagree, 3 = neither agree nor disagree, 4 = somewhat agree, 5 = strongly agree. ^b^ Likert scale on time required to complete handoff per patient 1–5: 1 ≤ 2 min, 2 = 2–4 min, 3 = 5–10 min, 4 = 11–20 min, 5 ≥ 20 min. ^c^ Likert scale on provider satisfaction 1 = much worse, 2 = somewhat worse, 3 = neutral, 4 = somewhat better, 5 = much better. +I-PASS: Illness Severity, Patient Summary, Action List, Situational Awareness, Contingency Plan, and Synthesis by the receiver # See: Patients assigned to see that day, especially those with high symptom burden and need for medication adjustments. * Skip: Patients with low symptom burden but still admitted for acute illness are seen every 3–4 days.

## Data Availability

Due to institutional compliance policy, the data presented in this study are available on request from the corresponding author.
